# Interferon Regulatory Factor-5 Deficiency Ameliorates Disease Severity in the MRL/lpr Mouse Model of Lupus in the Absence of a Mutation in DOCK2

**DOI:** 10.1371/journal.pone.0103478

**Published:** 2014-07-30

**Authors:** Kei Yasuda, Amanda A. Watkins, Guneet S. Kochar, Gabriella E. Wilson, Bari Laskow, Christophe Richez, Ramon G. Bonegio, Ian R. Rifkin

**Affiliations:** Renal Section, Department of Medicine, Boston University School of Medicine, Boston, Massachusetts, United States of America; Pavillon Kirmisson, France

## Abstract

Interferon regulatory factor 5 (IRF5) polymorphisms are strongly associated with an increased risk of developing the autoimmune disease systemic lupus erythematosus. In mouse lupus models, IRF5-deficiency was shown to reduce disease severity consistent with an important role for IRF5 in disease pathogenesis. However these mouse studies were confounded by the recent demonstration that the IRF5 knockout mouse line contained a loss-of-function mutation in the dedicator of cytokinesis 2 (DOCK2) gene. As DOCK2 regulates lymphocyte trafficking and Toll-like receptor signaling, this raised the possibility that some of the protective effects attributed to IRF5 deficiency in the mouse lupus models may instead have been due to DOCK2 deficiency. We have therefore here evaluated the effect of IRF5-deficiency in the MRL/lpr mouse lupus model in the absence of the DOCK2 mutation. We find that IRF5-deficient (IRF5^−/−^) MRL/lpr mice develop much less severe disease than their IRF5-sufficient (IRF5^+/+^) littermates. Despite markedly lower serum levels of anti-nuclear autoantibodies and reduced total splenocyte and CD4^+^ T cell numbers, IRF5^−/−^ MRL/lpr mice have similar numbers of all splenic B cell subsets compared to IRF5^+/+^ MRL/lpr mice, suggesting that IRF5 is not involved in B cell development up to the mature B cell stage. However, IRF5^−/−^ MRL/lpr mice have greatly reduced numbers of spleen plasmablasts and bone marrow plasma cells. Serum levels of B lymphocyte stimulator (BLyS) were markedly elevated in the MRL/lpr mice but no effect of IRF5 on serum BLyS levels was seen. Overall our data demonstrate that IRF5 contributes to disease pathogenesis in the MRL/lpr lupus model and that this is due, at least in part, to the role of IRF5 in plasma cell formation. Our data also suggest that combined therapy targeting both IRF5 and BLyS might be a particularly effective therapeutic approach in lupus.

## Introduction

Systemic lupus erythematosus (SLE) is an autoimmune disease characterized by the loss of tolerance to chromatin and ribonucleoproteins and the deposition of immune complexes in various organs [1]. The clinical presentation is heterogeneous but disease manifestations may be severe. The treatment itself may cause appreciable morbidity and responses to treatment are often incomplete.

SLE is caused by an incompletely understood interaction between genetic and environmental factors [1]. Polymorphisms in the transcription factor interferon regulatory factor 5 (IRF5) have been strongly associated with an increased risk of developing lupus in multiple human genetic studies [2], [3], [4], [5]. The polymorphisms contributing to the high-risk IRF5 haplotype are thought to induce novel IRF5 isoforms and/or increase the level of IRF5 expression by increasing the stability of IRF5 protein or mRNA leading to IRF5 gain-of-function [4], [6], [7], [8], [9], [10], [11].

IRF5 plays an important role in TLR signaling through the induction of pro-inflammatory cytokines, type I interferons, chemokines and class switching to IgG2a [12], [13], [14]. The degree to which IRF5 plays a role in these TLR-induced responses is ligand-, cell type- and cytokine- dependent but IRF5 appears to be consistently involved in many TLR7- and TLR9-driven responses [15], [16], [17], [18]. As dysregulated TLR signaling, particularly through TLR7 and TLR9, may contribute to lupus pathogenesis it is possible that the effects of IRF5 in lupus are mediated through alterations in the strength or nature of TLR signaling events. IRF5 also participates in apoptotic pathways induced by viral infection, DNA damage, Fas ligand and TNF-related apoptosis-inducing ligand (TRAIL) [17], [19], [20]. Given the association of abnormal apoptosis regulation to lupus pathogenesis it is possible that IRF5 might also impact lupus pathogenesis through this mechanism.

A number of groups have examined the role of IRF5 in mouse models of SLE using IRF5 knockout mice. We found that IRF5 was critical for disease development in the FcγRIIB^−/−^Yaa and FcγRIIB^−/−^ lupus models as IRF5-deficient FcγRIIB^−/−^Yaa and FcγRIIB^−/−^ mice developed minimal disease manifestations [21]. Even deficiency of a single IRF5 allele conferred substantial protection as IRF5 heterozygous mice developed very little disease. Subsequent studies showed that IRF5 was also important for disease development in the MRL/lpr and pristane models of lupus [14], [22], [23], [24]. However, it was recently reported that the IRF5 knockout mouse line contains a mutation in the dedicator of cytokinesis 2 (DOCK2) gene [18], [25]. This mutation causes unstable DOCK2 mRNA expression resulting in very low DOCK2 protein expression [25]. Previous studies in DOCK2 knockout (DOCK2^−/−^) mice have shown that DOCK2 has important functions in the immune system. Trafficking of T cells, B cells and neutrophils is impaired in DOCK2^−/−^ mice due to defective chemokine receptor signaling [26], [27]. DOCK2^−/−^ mice develop enhanced T helper cell type 2 (Th2) responses because DOCK2-deficient CD4^+^ T cells cannot down-regulate surface IL-4 receptor α expression [28]. Plasmacytoid dendritic cells (pDCs) from DOCK2^−/−^ mice have a reduced capacity to produce IFN-α and IFN-β in response to TLR7 and TLR9 ligands [29]. The DOCK2 mutation present in IRF5^−/−^ C57BL/6 mice has been shown to cause a marked reduction in splenic marginal zone and mature B cell numbers, to enhance Th2-type IgG production and to affect certain aspects of TLR signaling [18], [25].

Given the effects of DOCK2 on immune function noted above, it is possible that if the DOCK2 mutation was present in the mouse lupus models studied then some of the protective effects attributed to IRF5 deficiency may instead have been due to DOCK2 deficiency. We have screened archived tail DNA using a PCR that we recently developed to specifically detect the DOCK2 mutation and found that many of the FcγRIIB^−/−^Yaa and FcγRIIB^−/−^ mice in our previous study contained the DOCK2 mutation [18]. In this report we have examined the role of IRF5 in the MRL/lpr lupus model using mice without the DOCK mutation. We have also specifically examined the effect of deficiency of a single allele of IRF5 in MRL/lpr, as we previously observed a marked protective effect of IRF5 heterozygosity in the FcγRIIB^−/−^Yaa and FcγRIIB^−/−^ models.

## Materials & Methods

### Ethics statement

Animal experiments were approved by the Institutional Animal Care and Use Committee at Boston University.

### Mice

IRF5^−/−^ mice backcrossed eight generations to C57BL/6 were obtained from T. Taniguchi (University of Tokyo, Tokyo, Japan) and T. Mak (University of Toronto, Toronto, Ontario, Canada) [12]. IRF5^−/−^ mice were backcrossed 7 generations to MRL/MpJ-Fas^lpr^ mice (Stock number 000485, Jackson, Bar Harbor, ME). Experimental mice were generated by breeding IRF5 heterozygous (IRF5^+/−^) MRL/lpr mice to obtain IRF5^+/+^, IRF5^+/−^ and IRF5^−/−^ littermates. Only female mice were used for this study.

Animal experiments were approved by the Institutional Animal Care and Use Committee at Boston University. Mice were housed in a Specific Pathogen Free facility using an individual ventilated caging system with nestlets or igloos as environmental enrichment. Mice had free access to food (irradiated diet) and water (Hydropac water source system).

Boston University has a strict humane endpoints policy in place for animal experiments (http://www.bu.edu/orccommittees/iacuc/policies-and-guidelines/humane-endpoints-policy/). We adhered strictly to this policy and used humane end points in all experiments. The specific criteria used to determine when the animals should be humanely euthanized included the following: swollen lymph nodes; dermatitis associated with skin ulceration or any degree of dermatitis associated with significant discomfort as manifested by persistent or frequent scratching or rubbing of the involved area; decreased mobility; ruffled fur coat or lack of grooming behavior; decreased food intake; hunched posture; respiration that was increased, decreased or appeared labored; inability to reach food or water; unsteady gait; weight loss; evidence of pain or discomfort. The mice were monitored daily by experienced full-time animal technicians under the direct supervision of qualified veterinarians. If the mice exhibited any of these criteria then they were immediately euthanized. The mice were euthanized by CO_2_ inhalation as the primary method of euthanasia followed by cervical dislocation as the secondary method of euthanasia consistent with the American Veterinary Medical Association Guidelines on Euthanasia. Analgesics and anesthetics were not administered, as the mice were euthanized if they exhibited evidence of pain or discomfort.

### PCR

**Figure 1:**
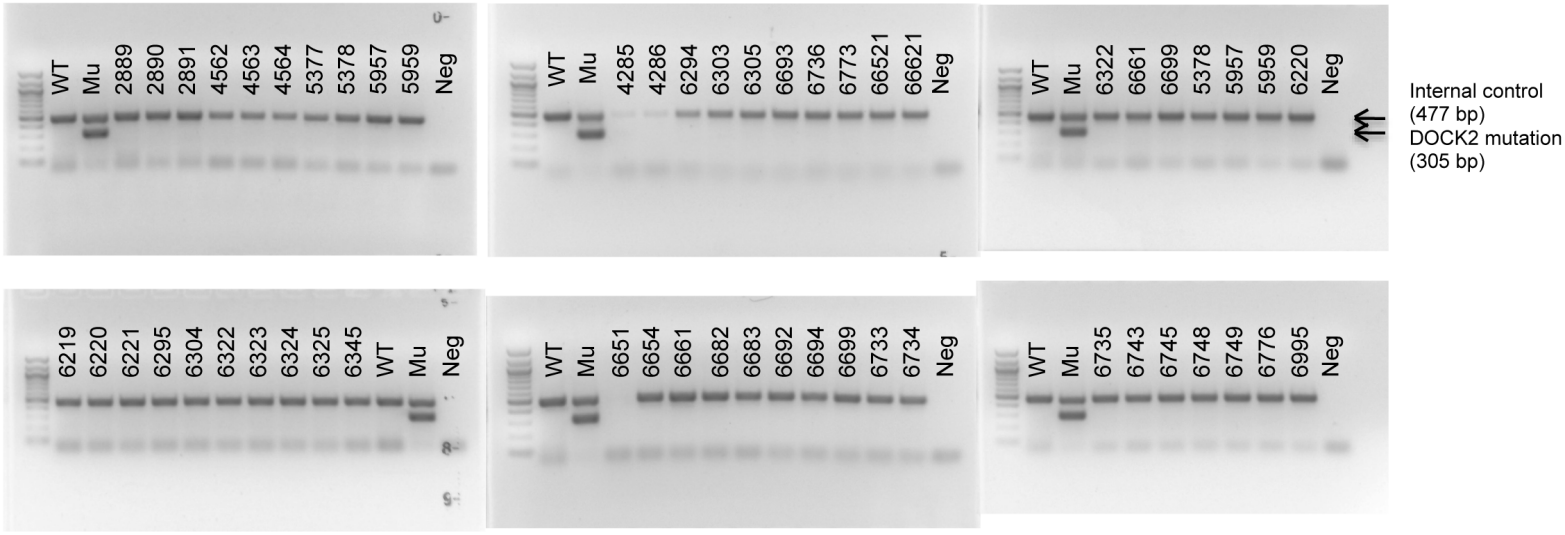
The experimental mice used in this study did not have the DOCK2 mutation. PCR to detect the DOCK2 mutation was performed on tail DNA of all mice used in this study. Experimental mice are indicated by number. DNA from a mouse with the DOCK2 mutation (Mu) was used as a positive control and gave a PCR product for the DOCK2 mutation (305 bp). DNA from a mouse without the DOCK2 mutation (WT) was used as a negative control. CD19 PCR (477 bp) was used as an internal control to verify the adequacy of DNA preparation in each sample.

The PCR used to detect the DOCK2 mutation was described previously [18]. PCR primers to detect the DOCK2 mutation are; 5′-GAC CTT ATG AGG TGG AAC CAC AAC C-3′ and 5′-GAT CCA AAG ATT CCC TAC AGC TCC AC-3′. The PCR detects the DOCK2 mutation as a 305-bp product. PCR primer sequences that recognize the CD19 gene were obtained from the Web site of The Jackson Laboratory (http://jaxmice.jax.org/strain/004126.html), and the primers used as an internal control to ensure that DNA preparation was adequate in all tail samples. All experimental MRL/mice were confirmed to have only wild type DOCK2 (Fig. 1).

### Flow cytometry

Splenocytes and bone marrow cells were treated with red blood cell lysing buffer (Sigma, St Louis, MO) to remove red blood cells. Splenocytes were labeled with monoclonal antibodies specific for CD4, CD8 and CD3 to identify T cell populations, with CD19 and B220 to identify B cells, and with CD19, B220, AA4.1, IgM, CD21 and CD23 to identify spleen B cell subsets. The bone marrow cells were stained with B220, AA4.1 and IgM to identify bone marrow B cell subsets. For staining of spleen plasmablasts, antibodies against CD3, CD19, CD22, B220, CD44 and CD138 were used. For staining of bone marrow plasma cells, antibodies against CD4, CD8, Gr-1 and F4/80 were used to gate out non-B cells, and plasma cells (CD138^+^B220^−^) were identified using antibodies against B220 and CD138. All antibodies were from either BD Biosciences (San Jose, CA) or eBioscience (San Diego, CA). Immunoflourescence was measured with a FACScan flow cytometer or LSRII (BD Bioscience). The data was analyzed using FlowJo software (Tree Star, Ashland, OR).

### Serological assays

IgG isotypes and IgM were measured by ELISA established using antibodies from BD Biosciences (San Jose, CA) and SouthernBiotech (Birmingham, AL). Anti-nuclear autoantibody (ANA) titer was measured by immunofluorescence using HEp-2-coated-slides (Antibodies Incorporated, Davis, CA). Anti-double stranded (ds) DNA autoantibodies were measured by immunofluorescence analysis of Crithidia lucillae kinetoplast staining (Antibodies Incorporated, Davis, CA). Blood urea nitrogen (BUN) levels were measured using a QuantiChrom Urea Assay kit (BioAssay Systems, Hayward, CA). Serum BLyS levels were measured by ELISA developed using antibodies from R&D Systems (Minneapolis, MN).

### Histology

Hematoxylin and eosin-stained kidney sections were evaluated in a blinded manner. Randomly selected areas of cortex were digitally photographed by a blinded investigator using an RT color spot camera (Diagnostic Instruments, Sterling Heights, MI) and Spot Advanced software version 4.0.9 (Diagnostic Instruments). Fifty glomeruli from each animal were examined to determine the percentage of glomeruli with severe lesions (glomerular crescents or areas of necrosis), mild lesions (mesangial expansion) or no lesions. Crescents were identified by the presence of 3 palisading layers of cells in the extra-capillary space and fibrinoid necrosis was identified by its classic eosinophillic and acellular appearance. Mesangial expansion was defined as prominence of the mesangial matrix as compared to normal glomeruli.

### Statistical analysis

In all cases groups were compared using a Kruskal-Wallis analysis of variance on ranks because some of our data (e.g. the ANA titers) are not normally distributed. In experiments where there were significant differences between groups (P<0.05), the Holm-Sidak method was employed for *post-hoc* analysis and to correct for multiple comparisons. The Kaplan-Meier method with log-rank statistic was used to analyze the survival studies. All statistics were performed using SigmaPlot 12.5 (Systat, San Jose, USA) with P<0.05 considered as statistically significant.

## Results

### Experimental mice used in this study did not have the DOCK2 mutation

In this study we wanted to avoid as far as possible the presence of factors other than IRF5 expression itself that might alter disease development, including differences in the microbiome, genetic differences between independently maintained mouse lines, and, in particular, the presence of the DOCK2 mutation. We therefore generated the experimental groups by breeding IRF5 heterozygous (IRF5^+/−^) MRL/lpr mice to generate IRF5-sufficient (IRF5^+/+^), IRF5-heterozygous (IRF5^+/−^) and IRF5-deficient (IRF5^−/−^) littermates. Only female littermates were used and were kept together in the same cage until the termination of the experiment at 16 weeks of age. We previously developed a genomic PCR to detect the DOCK2 mutation [18] and we used this PCR to determine whether the experimental mice had the DOCK2 mutation. As shown in Fig. 1, none of these mice had the DOCK2 mutation.

### Lymphoid tissue size and splenic T cell number are reduced in IRF5-deficient MRL/lpr mice, whereas the percentage of splenic B cells is increased

**Figure 2:**
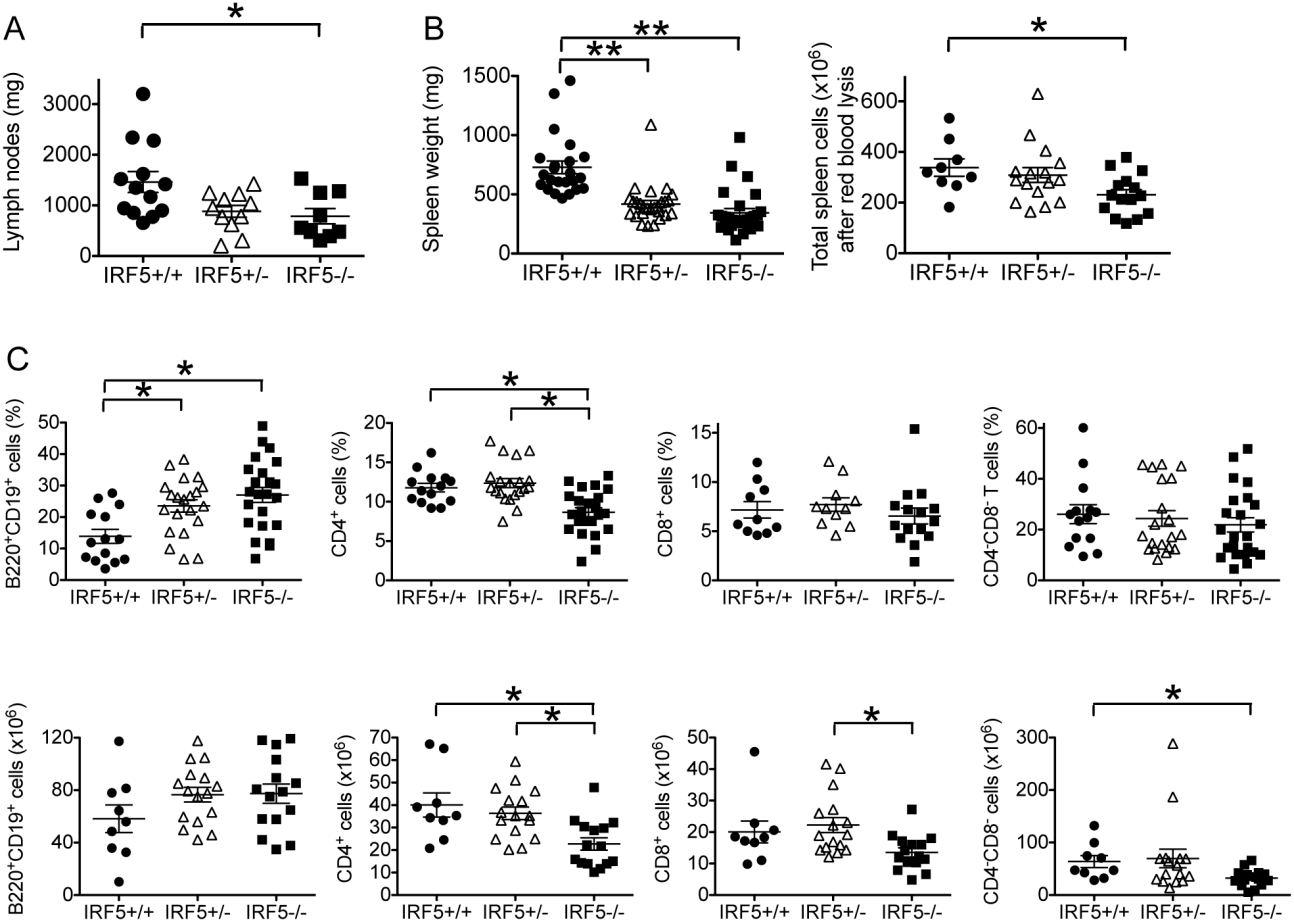
Lymphadenopathy, splenomegaly and splenic CD4^+^ T cell numbers are reduced in IRF5-deficient MRL/lpr mice. A. Lymph node weight and B. Spleen weight and splenocyte number in IRF5^+/+^, IRF5^+/−^ and IRF5^−/−^ female MRL/lpr littermates. C. Percentages (upper panels) and total number (lower panels) of splenic B cells and T cell populations in IRF5^+/+^, IRF5^+/−^ and IRF5^−/−^ female MRL/lpr littermates analyzed by flow cytometry. Mice were analyzed at 16 weeks of age. Bars represent mean ± SEM. *p<0.05; **p<0.01.

Lymphadenopathy and splenomegaly are prominent features of murine lupus models and, as expected, this was observed in the IRF5^+/+^ MRL/lpr mice. The degree of lymphadenopathy and splenomegaly was substantially reduced in the IRF5^−/−^ MRL/lpr mice with a concomitant reduction in total spleen cell number (Fig. 2A, B). The reduction in spleen cell number was due largely to reductions in the number of CD4^+^ T cells and CD4^−^CD8^−^ (double negative) T cells although there was also a trend towards a reduction in CD8^+^ T cells (Fig. 2C). In contrast, the percentage of splenic B cells was approximately twice as high in the IRF5^−/−^ MRL/lpr mice as compared to the IRF5^+/+^ MRL/lpr mice although there was no statistically significant difference in total B cell numbers between the groups. Thus, IRF5 plays an important role in regulating splenic T cell numbers, but not B cell numbers, in MRL/lpr mice.

### Serum autoantibody levels and Th1-driven IgG isotypes are reduced in IRF5-deficient MRL/lpr mice

**Figure 3:**
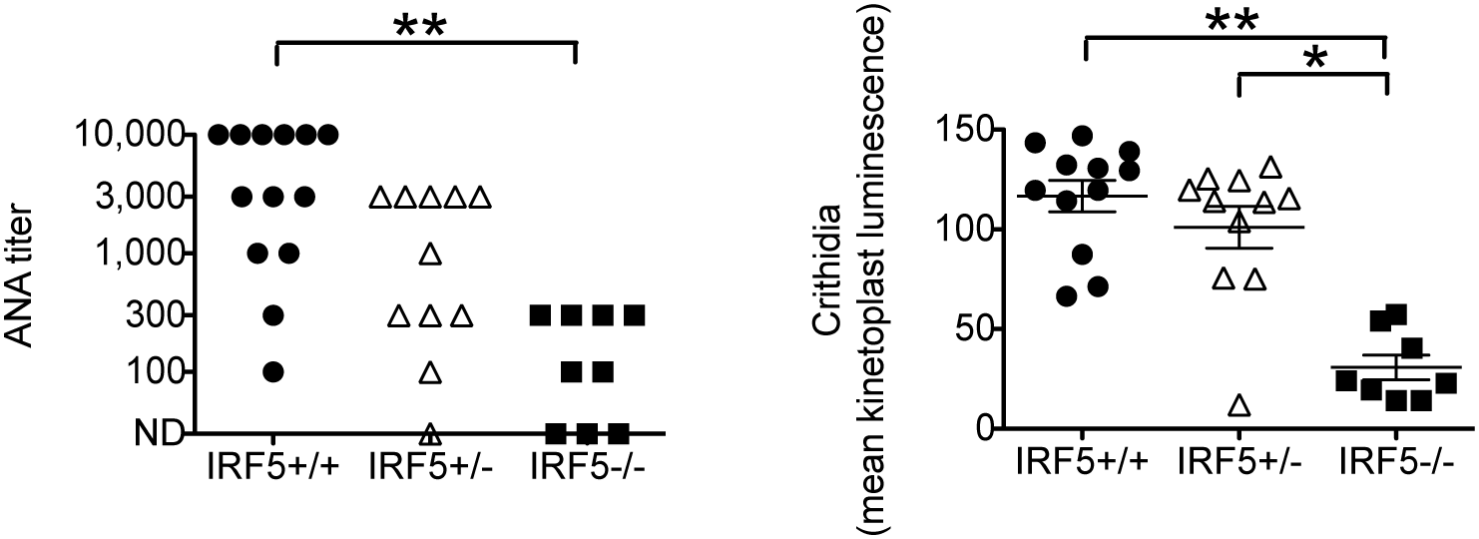
Decreased autoantibody production in IRF5-deficient MRL/lpr mice. Sera from IRF5^+/+^, IRF5^+/−^ and IRF5^−/−^ female MRL/lpr littermates were analyzed at 16 weeks of age. Anti-nuclear antibody (ANA) titers were measured by HEp2 cell immunofluorescence (left-hand panel) and anti-double stranded DNA (dsDNA) antibodies were measured by Crithidia lucillae luminescence (right-hand panel). Bars represent mean ± SEM. *p<0.05; **p<0.01.

Autoantibodies against nuclear antigens are a characteristic feature of SLE and likely contribute to disease pathogenesis [1]. Most IRF5^+/+^ MRL/lpr mice had high serum levels of anti-nuclear antibodies (ANA) as measured by immunofluorescence on HEp2 cells whereas ANA levels were markedly reduced in the IRF5^−/−^ MRL/lpr mice (Fig. 3). Similarly, anti-double-stranded (ds) DNA autoantibody levels, as measured by Crithidia immunofluorescence, were much lower in IRF5^−/−^ MRL/lpr mice (Fig. 3).

**Figure 4:**
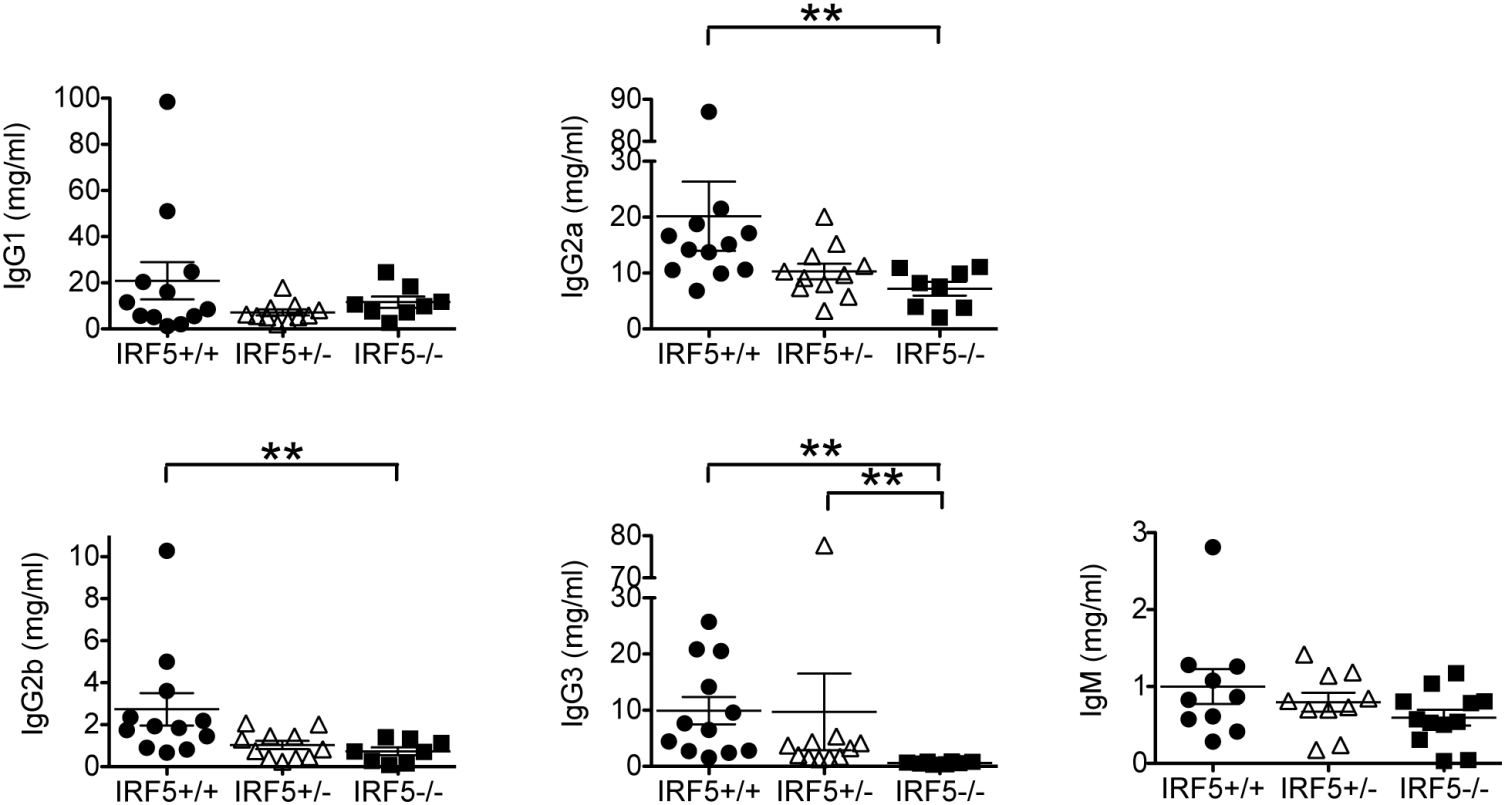
Decreased serum IgG2a, IgG2b and IgG3 levels in IRF5-deficient MRL/lpr mice. Sera from IRF5^+/+^ (n = 12), IRF5^+/−^ (n = 11) and IRF5^−/−^ (n = 8) female MRL/lpr littermates were analyzed at 16 weeks of age. IgG and IgM levels were measured by ELISA. Bars represent mean ± SEM. **p<0.01.

In terms of serum IgG isotypes, IgG2a levels were lower in IRF5^−/−^ MRL/lpr mice as compared with IRF5^+/+^ MRL/lpr mice (Fig. 4). This was expected as IRF5 has been shown to induce IgG2a production by directly binding to the IgG2a locus [14]. However levels of IgG2b and IgG3, but not of IgG1 or IgM, were also lower in IRF5^−/−^ MRL/lpr mice suggesting that IRF5 may regulate IgG isotype production in MRL/lpr mice not only through direct binding to the IgG2a locus but also through the generation of a Th1-like immune response.

### Renal disease is less severe in IRF5-deficient MRL/lpr mice

**Figure 5:**
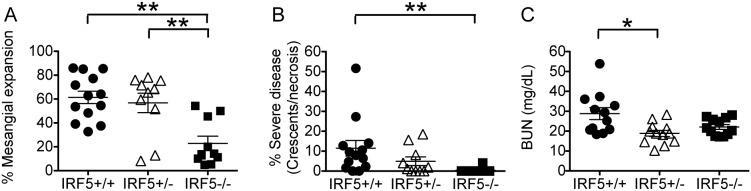
Less severe renal disease in IRF5-deficient MRL/lpr mice. A and B, Kidneys from IRF5^+/+^ (n = 13), IRF5^+/−^ (n = 10) and IRF5^−/−^ (n = 10) female MRL/lpr littermates were analyzed at 16 weeks of age. Renal disease was quantified by measuring the percentage of glomeruli in each mouse showing evidence of mesangial expansion (A), and the percentage of glomeruli in each mouse with crescents or necrosis (B). C, Serum BUN levels in IRF5^+/+^ (n = 12), IRF5^+/−^ (n = 12) and IRF5^−/−^ (n = 12) female MRL/lpr littermates at 16 weeks of age. Bars represent mean ± SEM. *p<0.05; **p<0.01.

Mesangial matrix expansion or mesangial hypercellularity are features of mild human lupus nephritis (class II) whereas glomerular crescents and necrosis are features of more severe proliferative lupus nephritis (class III or IV) [30]. All IRF5^+/+^ MRL/lpr mice exhibited a moderate to high degree of mesangial expansion with much less mesangial expansion being seen in IRF5^−/−^ MRL/lpr mice (Fig. 5A). Approximately half the IRF5^+/+^ MRL/lpr mice had developed severe renal disease by the end-point of the study at 16 weeks of age as evidenced by the presence of glomerular crescents or necrosis. In contrast, proliferative lupus nephritis was not observed on histological examination in IRF5^−/−^ MRL/lpr mice (Fig. 5B). Functional renal failure is characterized by an elevation in serum blood urea nitrogen (BUN) levels with BUN levels starting to rise when more than 50% of renal function has been lost [31]. The serum BUN level in non-autoimmune 16-week-old female C57BL/6 mice is approximately 22 mg/dl (http://jaxmice.jax.org/support/phenotyping/B6data000664.pdf). The highest serum BUN levels were seen in the IRF5^+/+^ MRL/lpr mice with 5 of the 12 mice, having serum BUN levels greater than 30 mg/dl indicative of marked renal failure (Fig. 5C). However, approximately half the IRF5^+/+^ MRL/lpr mice had less severe renal disease with serum BUN levels either normal or modestly elevated. None of the IRF5^−/−^ MRL/lpr mice had serum BUN levels greater than 30 mg/dl, consistent with the absence of severe disease on renal histology.

### IRF5-deficient MRL/lpr mice survive longer than IRF5-sufficient MRL/lpr mice

**Figure 6:**
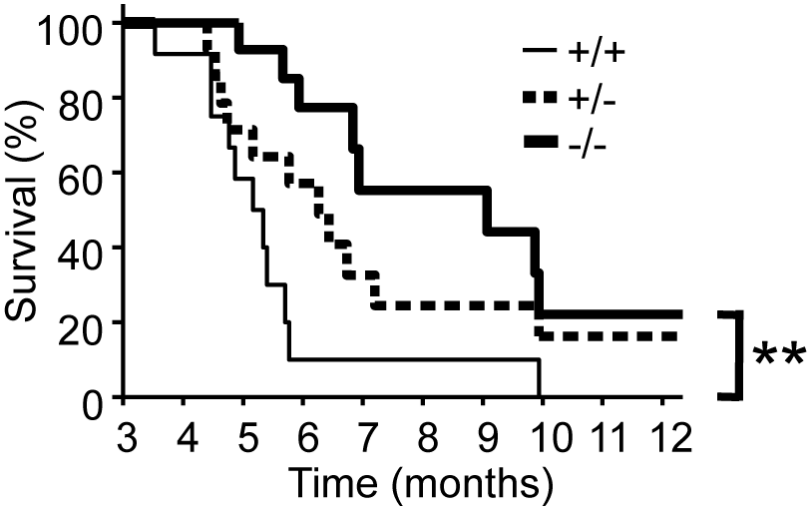
Increased survival in IRF5-deficient MRL/lpr mice. IRF5^+/+^ (n = 12), IRF5^+/−^ (n = 14) and IRF5^−/−^ (n = 14) female MRL/lpr littermates were observed until they met the criteria for euthanasia based on predetermined humane endpoints. ** p<0.01.

To determine whether the decrease in disease severity would translate into differences in survival, we bred new cohorts of female IRF5^+/+^, IRF5^+/−^and IRF5^−/−^ MRL/lpr littermates and monitored them until they met predetermined criteria for euthanasia. IRF5^+/+^ MRL/lpr mice had a median survival of 155 days, consistent with previous reports (Fig. 6) [32], [33]. The IRF5^−/−^ MRL/lpr littermates survived significantly longer with a median survival of 272 days (p<0.01).

### IRF5-heterozygote MRL/lpr mice are only modestly protected from disease

We had previously found in the FcγRIIB^−/−^Yaa and FcγRIIB^−/−^ lupus models that IRF5 heterozygote mice developed minimal clinical manifestations even though they still expressed approximately 40% as much IRF5 protein as IRF5-sufficient mice [21]. To determine whether loss of a single allele of IRF5 would have similar effects in the MRL/lpr model, we included IRF5 heterozygote littermates in all experiments. We found that IRF5^+/−^ MRL/lpr mice had smaller spleens and an increased percentage of splenic B cells as compared with IRF5^+/+^ littermates, but there were no statistically significant difference in T cell percentages, autoantibody production or serum IgG isotypes (Fig. 2, 3, 4). IRF5^+/−^ mice exhibited less severe end-organ disease in that IRF5^+/−^ mice had lower serum BUN levels and a trend toward longer survival (median = 188 days in IRF5^+/−^ MRL/lpr mice vs 155 days in IRF5^+/+^ MRL/lpr mice, p = 0.07), however the increase in survival was modest as compared with IRF5^−/−^ littermates which lived to a median of 272 days (Fig. 5 and 6). Although the serum BUN levels in the IRF5^+/−^ MRL/lpr mice appear lower than in the IRF5^−/−^ MRL/lpr mice, this is because there were a number of mice in the IRF5^+/−^ MRL/lpr group with particularly low BUN levels. This likely represents random variability of BUN within the normal range and there is no statistical difference between these groups (p = 0.82).

### IRF5 does not regulate the production of B lymphocyte stimulator in MRL/lpr mice

**Figure 7:**
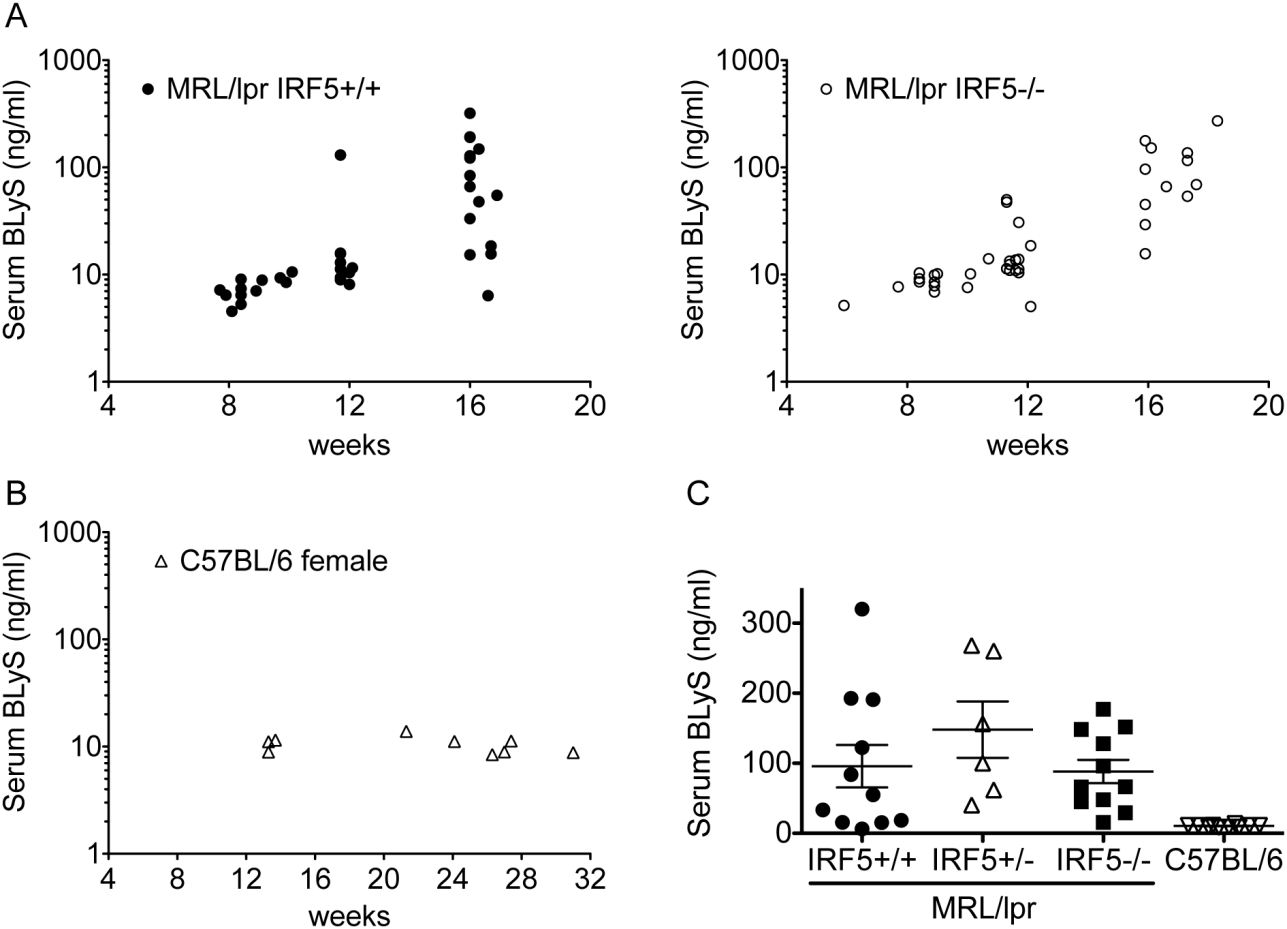
Serum BLyS levels are elevated in MRL/lpr mice but are not affected by IRF5 deficiency. A and B. BLyS levels in the sera of IRF5^+/+^ MRL/lpr mice and IRF5^−/−^ MRL/lpr mice (A), and C57BL/6 mice (B) at several ages were measured by ELISA. C. BLyS levels in the sera of sixteen week old IRF5^+/+^ (n = 11), IRF5^+/−^ (n = 6) and IRF5^−/−^ (n = 11) female MRL/lpr mice were measured by ELISA. The BLyS serum levels of the C57BL/6 mice (n = 10) shown in figure 7B (age range 13–32 weeks) are included for data comparison. Bars represent mean ± SEM. No significant differences were found between any of the MRL/lpr experimental groups.

B lymphocyte stimulator (BLyS, also known as BAFF) is a cytokine belonging to the TNF family that is thought to play an important role in the pathogenesis of human lupus [34]. BLyS overexpression causes a lupus-like disease in mice by promoting TLR7/9 expression in B cells and the TLR-induced production of autoantibodies [35]. As TLR7/8 activation can induce BLyS expression [36] and IRF5 has been implicated in BLyS production [37], we investigated whether IRF5 might regulate BLyS production in MRL/lpr mice. Serum BLyS levels increased markedly in MRL/lpr mice with age (Fig. 7A), consistent with previous reports [38], whereas serum BLyS levels did not change with age in C57BL/6 mice (Fig. 7B). Notably, serum BLyS levels in IRF5^+/−^ and IRF5^−/−^ MRL/lpr mice were no different from those in IRF5^+/+^ MRL/lpr mice (Fig. 7A and 7C). Thus IRF5 does not regulate BLyS production in MRL/lpr mice.

### Role of IRF5 in B cell development in MRL/lpr mice

**Figure 8:**
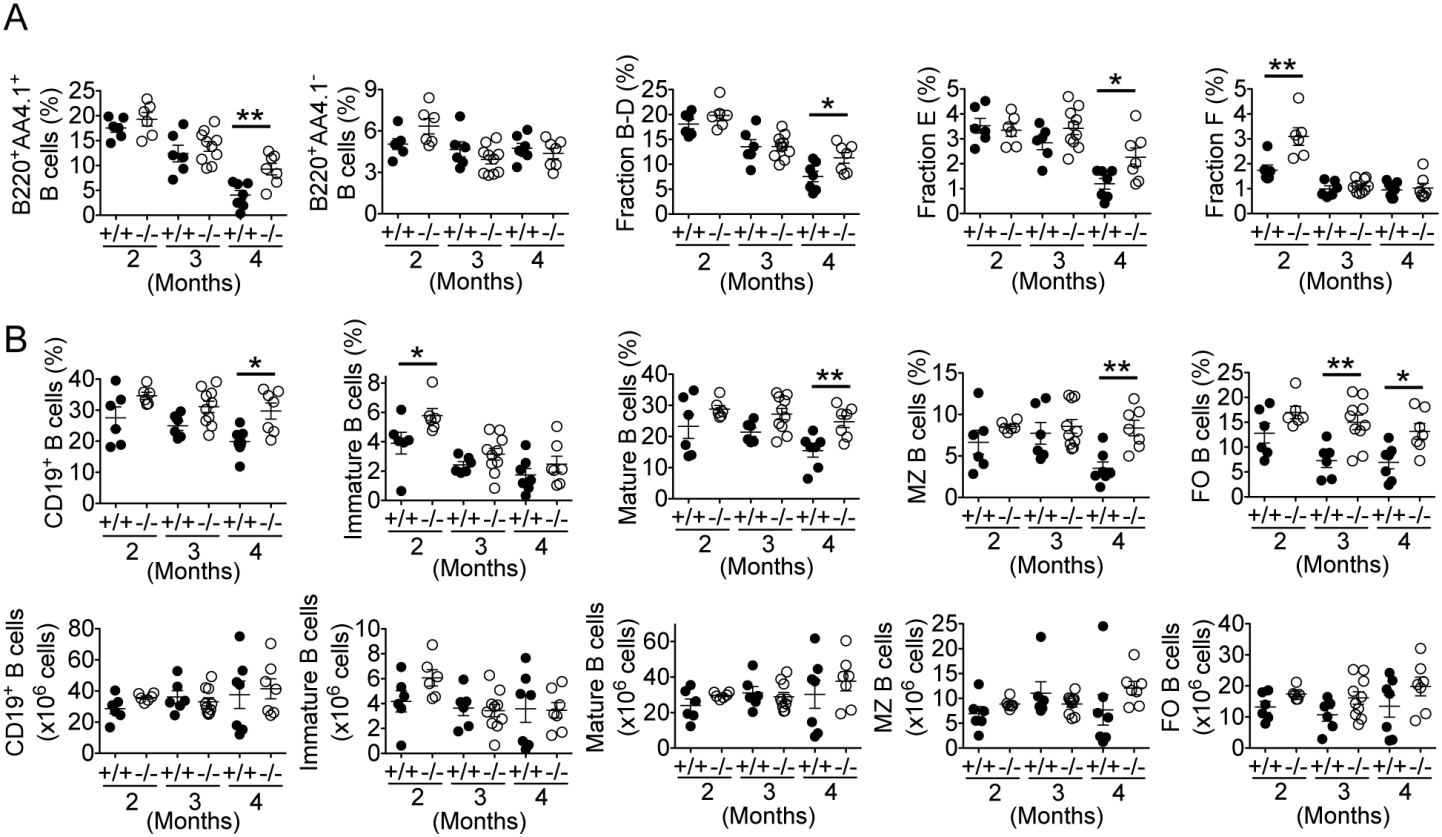
B cell development in IRF5^+/+^ and IRF5^−/−^ MRL/lpr mice. Bone marrow (A) and spleen cells (B) from 2, 3, and 4-month-old IRF5^+/+^ (filled circles) and IRF5^−/−^ (open circles) MRL/lpr mice were stained with antibodies against B220, AA4.1, IgM, CD19, CD21 and CD23. A. In bone marrow, percentages of B220^+^AA4.1^+^ (pro-B, pre-B and immature B) and B220^+^AA4.1^−^ (mature B) cells, percentages of Hardy fractions B-D (pro-B and pre-B; B220^+^IgM^−^), fraction E (immature B; B220^intermediate^IgM^+^), and fraction F (mature or re-circulating B; B220^high^IgM^+^) were determined. B. In spleen, percentages and cell numbers of B cells (CD19^+^), immature B cells (B220^+^AA4.1^+^) and mature B cells (B220^+^AA4.1^−^) were determined. Mature B cells were further classified as marginal zone (MZ) B cells (CD21^+^CD23^low^) and follicular (FO) B cells (CD23^+^) based on CD21 and CD23 expression. Each dot represents an individual mouse. Bars represent mean ± SEM. *p<0.05; **p<0.01.

Young IRF5-deficient C57BL/6 mice without the DOCK2 mutation exhibit normal B cell development [18], [25]. However, it has been reported that older IRF5-deficient non-autoimmune mice have a marked accumulation in the spleen of CD19^+^B220^−^ cells, a 6-fold increase in immature B cells and a decrease in plasma cells with a reduction in serum IgG1, IgG2a and IgG2b levels [39]. To determine whether the reduced serum IgG levels seen in the IRF5^−/−^ MRL/lpr mice were associated with a similar B cell developmental phenotype, and because we observed an increased percentage of CD19^+^ spleen B cells in the IRF5^−/−^ MRL/lpr mice, we performed a detailed analysis of B cell development in IRF5^+/+^ and IRF5^−/−^ MRL/lpr mice. In the bone marrow at 2 and 3 months of age IRF5^+/+^ and IRF5^−/−^ MRL/lpr mice had similar percentages of B220^+^AA4.1^+^ cells (pro-B, pre-B and immature B cells) and B220^+^AA4.1^−^ cells (mature B cells) (Fig. 8A). This was confirmed by analysis of Hardy fractions which showed that IRF5^+/+^ and IRF5^−/−^ MRL/lpr mice had similar percentages of Hardy fractions B-D (pro-B and pre-B cells; B220^+^IgM^−^) and fraction E (immature B cells; B220^intermediate^IgM^+^) although IRF5^−/−^ MRL/lpr mice had a higher percentage of fraction F (mature B cells or re-circulating B cells; B220^high^IgM^+^) at 2 months of age. By 4 months of age, the percentages of all bone marrow B cell subsets in both IRF5^+/+^ and IRF5^−/−^ MRL/lpr mice were generally lower than at the 2 month time point. However, at the 4 month time point, the percentages of B220^+^AA4.1^+^ cells, fraction B-D and fraction E in the IRF5^−/−^ MRL/lpr mice were higher than those in their IRF5^+/+^ MRL/lpr littermates although the percentages of B220^+^AA4.1^−^ mature B cells and fraction F were similar (Fig. 8A). In the spleen at 4 months of age, IRF5^−/−^ MRL/lpr mice had an increased percentage of CD19^+^ B cells compared to IRF5^+/+^ MRL/lpr littermates (Fig. 8B), consistent with the analysis in Fig. 2C. More detailed analysis showed that this was due to an increased percentage of mature B cells (both marginal zone and follicular) but not immature B cells (Fig. 8B upper panel). However, because total splenocyte number was lower in IRF5^−/−^ MRL/lpr mice, the total numbers of each B cell subset did not differ between IRF5^+/+^ and IRF5^−/−^ MRL/lpr mice (Fig. 8B lower panel). Overall, these results demonstrate that IRF5 does not play a significant role in B cell development, at least up until the splenic mature B cell stage, in the MRL/lpr lupus model.

### IRF5-deficient MRL/lpr mice have greatly reduced numbers of spleen plasmablasts and bone marrow plasma cells

**Figure 9:**
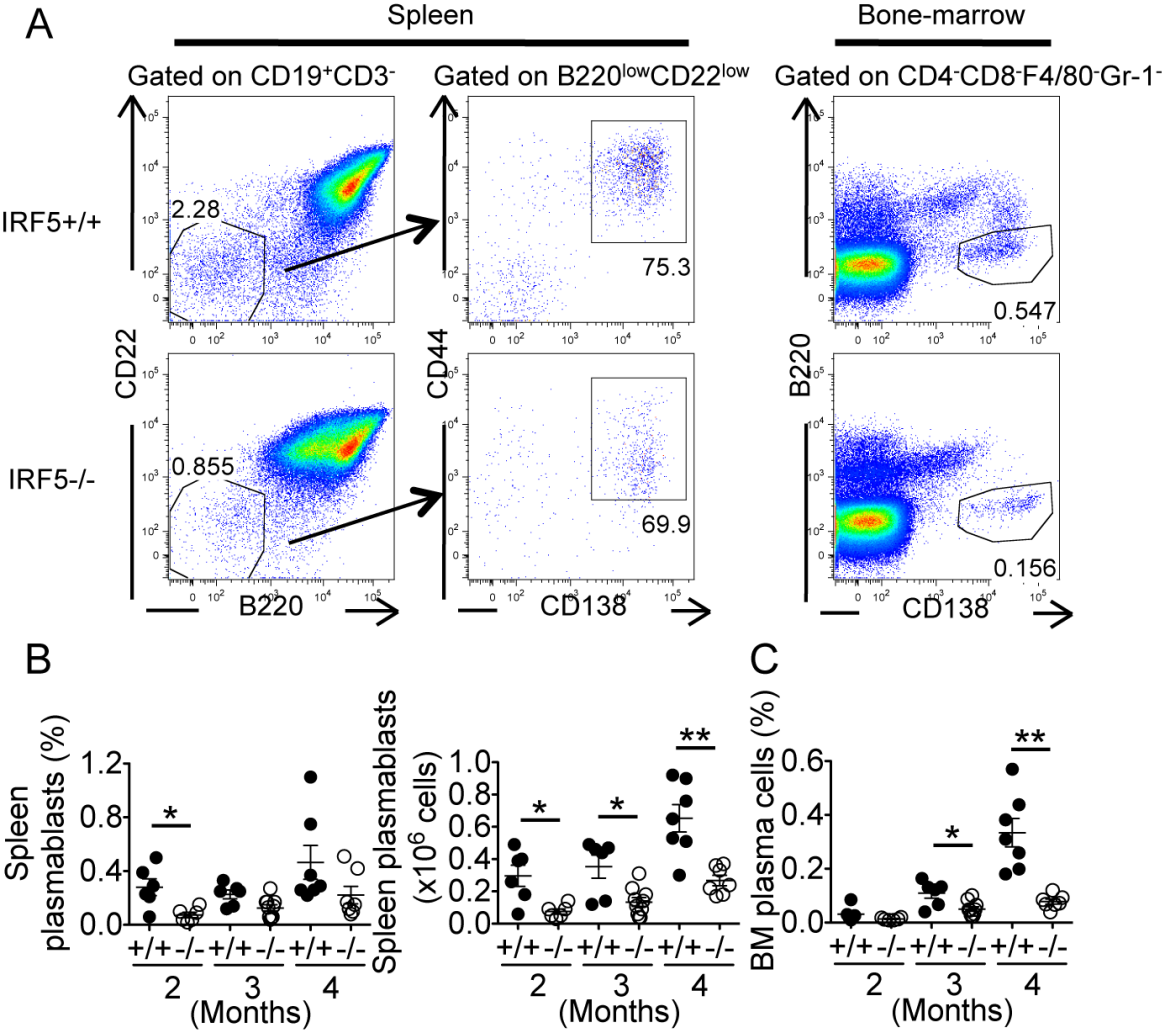
IRF5-deficient MRL/lpr mice have greatly reduced numbers of spleen plasmablasts and bone marrow plasma cells. A. Spleen cells from IRF5^+/+^ and IRF5^−/−^ MRL/lpr mice at 4 months of age were stained with antibodies against CD19, CD3, B220, CD22, CD44 and CD138. Plasmablasts (B220^low^CD22^low^CD44^+^CD138^+^) were identified within the splenic B cell (CD19^+^CD3^−^) population. Bone-marrow cells from IRF5^+/+^ and IRF5^−/−^ MRL/lpr mice were stained with antibodies against CD4, CD8, F4/80, Gr-1, B220 and CD138. CD4^−^CD8^−^F4/80^−^Gr-1^−^ cells were gated to analyze plasma cells (B220^−^CD138^+^). B. Numbers and percentages of spleen plasmablasts and bone marrow plasma cells of IRF5^+/+^ (filled circles) and IRF5^−/−^ (open circles) MRL/lpr mice at 2, 3 and 4 months of age are shown. Each dot represents an individual mouse. Bars represent mean ± SEM. *p<0.05; **p<0.01.

As IRF5^−/−^ MRL/lpr mice had much lower levels of autoantibody and immunoglobulin, especially IgG2a, IgG2b and IgG3 (Fig. 3 and 4), we hypothesized that IRF5 is involved in a further step of B cell activation or differentiation leading to the generation of plasmablasts and plasma cells. To test this, we examined plasmablasts in the spleen as well as plasma cells in the bone marrow of IRF5^+/+^ and IRF5^−/−^ MRL/lpr mice. High levels of splenic plasmablasts (CD19^+^B220^low^CD22^low^CD138^+^CD44^+^ cells) and bone marrow plasma cells (B220^−^CD138^+^ cells) were evident in IRF5^+/+^ MRL/lpr mice, particularly at 4 months of age (Fig. 9A and B). Notably however, splenic plasmablast numbers and bone marrow plasma cell percentages were both substantially lower in IRF5^−/−^ MRL/lpr mice at 4 months of age, with the reduction being evident in the spleen even at the 2 month time point. Thus IRF5 is required for optimal plasmablast and plasma cell formation in MRL/lpr mice.

## Discussion

The IRF5 knockout mouse line has been used by many investigators to determine the function of IRF5. It was recently reported that this line contains a loss-of-function mutation in the DOCK2 gene [25]. As DOCK2 plays an important role in immune function including in TLR signaling, lymphocyte trafficking and the development of Th2-type immune responses it is conceivable that certain effects previously attributed to IRF5 deficiency might instead have been due to the DOCK2 mutation. The effect of IRF5 deficiency in the MRL/lpr lupus model was reported before the DOCK2 mutation was discovered [22] and no information is available as to whether the DOCK2 mutation was present in the experimental mice in that study. One goal of our current study was to determine the effect of IRF5 in MRL/lpr mice in which the DOCK2 mutation was known to be absent.

We found that IRF5^−/−^ MRL/lpr mice developed substantially less severe disease than their IRF5^+/+^ MRL/lpr littermates. IRF5^−/−^ MRL/lpr mice had smaller spleens and lymph nodes, reduced titers of anti-nuclear and anti-dsDNA autoantibodies, lower levels of the complement-fixing IgG isotypes IgG2a, IgG2b and IgG3, less severe kidney disease and greatly improved survival. This protective effect of IRF5 deficiency is similar to that previously reported [22] although there are some differences. The IRF5^+/+^ MRL/lpr mice in our study had a more severe phenotype as judged by the length of survival with a median survival of 155 days as compared to about 220 days in the study by Tada et al. This may explain why the median survival of the IRF5^−/−^ MRL/lpr mice in our study was shorter (272 days versus greater than 350 days). Thus, IRF5 inhibition may be less effective when disease is more severe. We also observed a marked reduction in total serum IgG2a levels which was not seen in the study by Tada et al.

Another goal of this current study was to determine the effect of the loss of a single allele of IRF5 on disease pathogenesis in the MRL/lpr model. We had previously found that in the FcγRIIB^−/−^Yaa and FcγRIIB^−/−^ lupus models, the IRF5 heterozygotes were markedly protected from disease and exhibited a phenotype similar to the IRF5-deficient mice, even though IRF5 protein expression was still approximately 40% of the wildtype level [21]. In this current study we found that the IRF5^+/−^ MRL/lpr mice developed less severe disease than IRF5^+/+^ MRL/lpr littermates but the degree of protection was not as marked as was seen in the FcγRIIB^−/−^Yaa and FcγRIIB^−/−^ models. One possible explanation for this difference is that the pathogenesis of disease may differ somewhat in the different models with certain cell types playing a more important role in one model than the other. As the relative contribution of IRF5 to TLR signaling events is known to be cell-type and ligand-dependent, the level of IRF5 expression may have a more dramatic effect in some cell types than in others. Future studies with cell-specific IRF5 deletion may help to address this issue. Another possible explanation is that the combination of IRF5 heterozygosity and the DOCK2 mutation may have exerted a more protective effect than IRF5 heterozygosity alone. We are currently generating new cohorts of IRF5^+/+^, IRF5^+/−^ and IRF5^−/−^ FcγRIIB^−/−^Yaa and FcγRIIB^−/−^ mice without the DOCK2 mutation to definitively answer this question.

Although the total number of spleen cells was reduced in IRF5^−/−^ MRL/lpr mice as compared with IRF5^+/+^ MRL/lpr mice, the total number of splenic B cells was similar in the IRF5^−/−^ MRL/lpr mice. However, serum levels of anti-nuclear autoantibodies and the IgG isotypes IgG2a, IgG2b and IgG3 were greatly reduced in IRF5^−/−^ MRL/lpr mice. As IgG2a, IgG2b and IgG3 are all pathogenic isotypes in murine lupus [40], [41], [42], [43], IRF5 deficiency may ameliorate lupus at least in part through reduction of B cell antibody production. It is likely that this is due to a critical role for IRF5 in regulating MyD88-dependent antibody production downstream of TLR7 and TLR9, as the autoantibody and IgG isotype profiles are similar to those observed in MyD88-deficient MRL/lpr mice and TLR7/TLR9 double-deficient MRL/lpr mice [44].

The mechanisms through which IRF5 might regulate IgG class switching are incompletely understood. IRF5 regulates IgG2a class switching downstream of TLR9 in a B cell-intrinsic manner by directly binding to the IgG2a promoter [14]. However, the effects of IRF5 on IgG2b and IgG3 production suggest that additional mechanisms are operative. It is likely that these additional mechanisms apply only under certain activation conditions as, in healthy non-autoimmune C57BL/6 mice, IRF5 deficiency leads to reduced serum IgG2a levels but does not affect IgG2b or IgG3 levels [18]. Additional studies will be required to determine whether the dominant IRF5-dependent pathways regulating autoantibody production and IgG class-switching in vivo are B cell-intrinsic or B cell extrinsic.

To investigate possible mechanisms through which IRF5 might regulate autoantibody production, in addition to its role in IgG class switching, we studied the effect of IRF5 deficiency on B cell development in the MRL/lpr model. We found that the number of splenic CD19^+^ B cells as well as the numbers of immature, marginal zone and follicular B cells did not differ between IRF5^+/+^ and IRF5^−/−^ MRL/lpr mice. However, the number of splenic plasmablasts and bone marrow plasma cells were greatly reduced in the IRF5^−/−^ MRL/lpr mice. Thus IRF5 does not play a major role in B cell development up until the splenic mature B cell stage but is required for effective plasma cell generation in this autoimmune model.

The transition from mature B cell to plasma cell is a complex multistep process involving many cell types [45], [46], and IRF5 could potentially be involved in one or more steps of this process from B cell activation to final plasma cell formation. The relative importance of signals delivered through the B cell receptor or by T cells to plasma cell differentiation has not been resolved [45] and an important initial question to address is whether the role of IRF5 in plasma cell formation is B cell intrinsic or B cell extrinsic. B cell activation by TLR7 or TLR9 is known to induce IRF5-dependent B cell cytokine production [12], [18], and this initial activation of IRF5 could contribute to subsequent plasma cell formation. IRF5 has been shown to directly regulate the expression of Blimp-1, the master regulator of plasma cell differentiation, and it is possible that this could contribute to any B cell intrinsic effect of IRF5 [39], [47]. A separate issue that needs to be addressed is whether the markedly reduced number of plasma cells in the bone marrow of the IRF5^−/−^ MRL/lpr mice is due only to a reduction in plasma cell formation or whether it is also due to decreased survival of long-lived plasma cells in the bone marrow. IL-6 provides a crucial survival signal to bone marrow plasma cells [48] and IRF5 can regulate IL-6 production in a number of cell types [12], [18], [25], [49], [50].

Our finding that splenic CD4^+^ T cell numbers were substantially reduced in the IRF5^−/−^ MRL/lpr mice would be consistent with an important role for IRF5 in regulating T cell activation and/or survival. IRF5 in macrophages has been shown to promote Th1 and Th17 skewing following LPS activation [49]. It has also been reported that T cells express IRF5 in the presence of IFN-α [51], a cytokine thought to play a role in lupus pathogenesis [52]. Thus, IRF5 could potentially regulate T cell numbers either indirectly through effects on antigen-presenting cells or directly through a T cell intrinsic effect.

BLyS is thought to contribute to lupus pathogenesis and a BLyS inhibitor is now approved as a therapy for lupus [34]. We investigated whether IRF5 regulation of BLyS might partly explain the protective effect of IRF5 deficiency as splenocytes from IRF5-deficient non-autoimmune mice were shown to express lower levels of BLyS than IRF5-sufficient mice [37]. In addition, TLR7/8 can induce BLyS production from myeloid dendritic cells [36] and BLyS is able to induce class switch recombination [35], [53]. However, we found no difference in serum BLyS levels between IRF5^+/+^ and IRF5^−/−^ MRL/lpr mice with markedly elevated levels being found in both groups. This result indicates that the effects of IRF5 and BLyS on disease are mediated through independent pathways.

In summary, we have shown that IRF5 plays an important role in the development of disease in the MRL/lpr mouse model of lupus in the absence of the DOCK2 mutation and that IRF5 is required for the transition from mature B cells to plasma cells. Taken together with the human genetic data showing that gain-of-function polymorphisms of IRF5 are associated with an increased risk of developing lupus, this suggests that IRF5 may be a useful therapeutic target in lupus. In addition, the observation that IRF5 and BLyS contribute to disease pathogenesis through different mechanisms suggests the possibility that combined BLyS and IRF5 inhibition might be a more effective therapy for lupus than inhibition of either pathway alone.
